# Self-Organization, Entropy Generation Rate, and Boundary Defects: A Control Volume Approach

**DOI:** 10.3390/e23081092

**Published:** 2021-08-22

**Authors:** Jainagesh A. Sekhar

**Affiliations:** 1 MHI Inc., Cincinnati, OH 45215, USA; jainagesh.sekhar@uc.edu; 2 Department of Mechanical and Materials Engineering, University of Cincinnati, Cincinnati, OH 45221, USA

**Keywords:** self-organization, maximum entropy generation rate per unit volume, defects, solidification patterns, friction and wear textures, patterns, discontinuous self-organization, reorganization, utility of self-organization, dendrite spacing, auto correlation length

## Abstract

Self-organization that leads to the discontinuous emergence of optimized new patterns is related to entropy generation and the export of entropy. Compared to the original pattern that the new, self-organized pattern replaces, the new features could involve an abrupt change in the pattern-volume. There is no clear principle of pathway selection for self-organization that is known for triggering a particular new self-organization pattern. The new pattern displays different types of boundary-defects necessary for stabilizing the new order. Boundary-defects can contain high entropy regions of concentrated chemical species. On the other hand, the reorganization (or refinement) of an established pattern is a more kinetically tractable process, where the entropy generation rate varies continuously with the imposed variables that enable and sustain the pattern features. The maximum entropy production rate (MEPR) principle is one possibility that may have predictive capability for self-organization. The scale of shapes that form or evolve during self-organization and reorganization are influenced by the export of specific defects from the control volume of study. The control volume (CV) approach must include the texture patterns to be located inside the CV for the MEPR analysis to be applicable. These hypotheses were examined for patterns that are well-characterized for solidification and wear processes. We tested the governing equations for bifurcations (the onset of new patterns) and for reorganization (the fine tuning of existing patterns) with published experimental data, across the range of solidification morphologies and nonequilibrium phases, for metallic glass and featureless crystalline solids. The self-assembling features of surface-texture patterns for friction and wear conditions were also modeled with the entropy generation (MEPR) principle, including defect production (wear debris). We found that surface texture and entropy generation in the control volume could be predictive for self-organization. The main results of this study provide support to the hypothesis that self-organized patterns are a consequence of the maximum entropy production rate per volume principle. Patterns at any scale optimize a certain outcome and have utility. We discuss some similarities between the self-organization behavior of both inanimate and living systems, with ideas regarding the optimizing features of self-organized pattern features that impact functionality, beauty, and consciousness.

## 1. Introduction

In this article, we study the emergence of new steady state patterns, which discontinuously replace a previous steady state pattern, by a process called self-organization. Self-organization is the emergence of patterns displaying a unique order (e.g., crystal structure, elements of tessellations, dendritic patterns, fluid eddies, solar plasma winds, etc.). Self-organization in a system is enabled by processes that are internal to the system, as opposed to bringing order by new external constraints or forces that alter the system restraints. The trigger for self-organization may originate outside the system of study. Abrupt self-organization is not continuous reorganization. For chemical systems undergoing transformation, the continuous refinement of an existing pattern is referred to as the reorganization, or refinement, of a pattern. The continuous reorganization of the features of an existing pattern (e.g., cells becoming finer in a convective pattern) is commonly, smoothly enabled by smooth changes in the force driving the pattern formation. However, when the driving forces for reorganization become severe or exceed a threshold, a completely new order is suddenly experienced; this process is called self-organization [1,2,3,4,5,6,7].

Conditions can exist where the demands on the entropy production or entropy dissipation at the control volume boundary cause non-linearities in the derivative functions of the potential gradients that drive fluxes. Newer patterns can then arise from the amplifications of the perturbations that lead to a new type of order. Examples where such patterns are noted range from galaxy clusters, Hele-Shaw cells, natural convection, smokestacks, wear texture, evolutionary biological systems, and directional freezing [1,2,3,4,5,6,7,8,9,10,11,12,13,14,15,16,17,18,19,20,21,22,23,24,25,26,27,28,29,30,31,32,33,34,35,36,37,38,39,40,41,42,43,44,45,46,47,48,49,50,51,52,53,54,55,56,57,58,59,60,61,62,63,64,65,66,67,68,69,70,71,72,73,74,75].

The distinguishing feature of a system that is driven to self-organize is the demand on dissipation of entropy from a volume (control volume) to maintain a steady state [2]. The control volume is the volume of interest, where the process-driven conditions cause self-organization. Often, if not always, there is a production of entropy in that volume, which, in turn, may give rise to new internal boundaries, leading to new pattern formation and entropy transfer.

The reorganization or refinement of an established, self-organized structure (i.e., continuous reorganization) is often tractable by known kinetic equations of transport, with predetermined kinetic-rate coefficients [8]. Reorganization can be predicted by known kinetic coefficients, i.e., with measured values of material constants, such as the thermal conductivity or diffusion constants. The emergence of an entirely new self-organized pattern, on the other hand, is not as easily predictable because it involves comparisons between different distributions or competing reactions/morphologies across several length scales, without a clearly recognized governing principle for the selection process [1,2,3].

**Figure 1:**
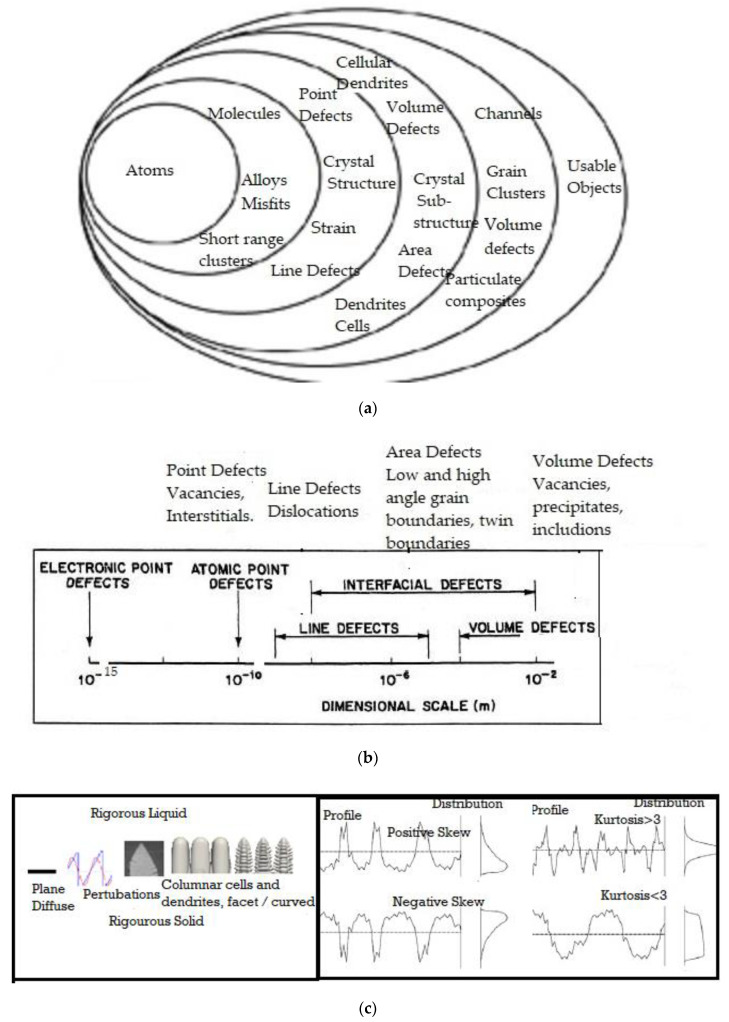
The typical length scales that are studied (**a**) for patterns and (**b**) for defects for metallurgical assessments of engineering properties, (**c**) shows the various shapes and arrays studied in this article for solidification on the left and for surface texture (with hierarchical nano features) on the right [56]. Note that the ripples at the peaks and valleys are also called nano-hierarchical structures depending on the scale.

Self-organization is a process in which patterns at a higher level (or length scale) of a system emerge from multi-scale interactions among the components of the system. Moreover, the rules specifying interactions among the system’s components are executed using only local information [1,2,3,4,5,6,7,8,9,10,11,12,13,14,15,16,17,18,75]. This is similar to the crystallizing of liquid into an ordered lattice (crystalline solid) during cooling, as opposed to simply cooling into a glassy state (studied in Section 4 of this article). As we will note below, patterns that form in microscopic and macroscopic systems, at a scale larger than the atomic-lattice-scale, are influenced by events that happen at the smaller lattice-scale. Self-organization leads to local order, which can happen across multiple length scales. Figure 1 describes the various scales that are studied, for a metallurgical assessment of engineering properties. We have chosen two examples for the quantitative study of self-organization from the metallurgical literature, namely solidification and friction/wear. In the solidification research-field, careful measurements are available for the scales and diving forces for various patterns, thus allowing for an analysis of the hypotheses in this article.

In this article, we reinforce the view that such an organization is a consequence of the maximum entropy production principle. This article reviews and extends the use of this principle for micrometer-scale patterns that are observed during directional solidification and solid-solid frictional surface interactions during wear. Directional solidification is chosen as the test scenario because such an experiment can isolate the accurately measurable or defined potential gradients. This is done in a manner, to control and drive the self-organization process, while minimizing the impact of any changes in the configurational entropy [1,18,19,20,21,22,23,24,25,26,27,28,29,30,31,32,33,34] of an array of similar patterns for the formulation of the problem. The possible surface texture reorganization during frictional contact of a pair of solid-solid objects, leading to wear, is discussed in Appendix A.

## 2. Formalism

Self-organization, reviewed by Tzafestas [4], is a feature noted in both inanimate and living systems. The processes that are associated with self-organization are feedback, encapsulation, autocatalysis, synchronization, critical-connectivity, and adaptation (a way to better adapt to the surroundings) [4,5,6]. Each of these features will be apparent for the directional solidification and the wear studies discussed in the next sections and Appendix A, respectively, although we will not relate processes directly to such nomenclature. The study of self-organization is also now linked with fields of study, such as beauty and complexity [7]. In this article, we will mostly be concerned with understanding the relationships between self-organization, pattern formation, and the entropy dissipation/production in a solidifying chemical system, i.e., with a phase change process occurring inside a defined and well-characterized control volume (CV) [2].

Inside a control volume, where self-organized patterns are studied, the variation of entropy per unit time can be divided into two parts, namely, the exchange of entropy with the environment and the internal entropy production/generation [1,2,3]. Such a balance can be written as:

d**S**e/dt + d**S**gen/dt = d**S**cv/dt (1)

where **S** is the entropy, d**S**cv/dt [2] is the accumulation/reduction of entropy per unit time inside the control volume (cv), d**S**e/dt is the flow of entropy per unit time between the environment and the system (recognized at the boundary of the control volume), and d**S**gen/dt is the internal entropy production rate inside the control volume. Energy and mass exchanges, because of potential gradients, lead to entropy production [1,2,3,4,5,6,7,8,9]. The d**S**eg/dt is a consequence of gradients of chemical potential, temperature, pressure, or reaction/transformation activity and their conjugate fluxes or flow of mass, heat, total-volume, and chemical-species, respectively [1,2,3,4,5,6,7,8,9].

### Steady State

In this article, we study systems at steady state. These could, of course, encompass the extremely time insensitive but highly non-equilibrium conditions at the control volume boundaries that experience very slow transitions to other states. For a system that is in a stationary or steady state, d**S**cv/dt = 0. A system that is in a steady state may not necessarily be in a state of *dynamic equilibrium* (equilibrium implies that d**S**gen/dt = 0), as several of the physical processes involved are not reversible, i.e., they produce entropy.

The concept of a steady state has relevance in many fields. If a system is in a steady state, then the recently observed behavior of the system will be the same in the future, as in the present. The generation of entropy is possible at various macro-, micro-, statistical, and quantum scales. A steady state is similar to a stationary quantum state with all measurable physical quantities remaining constant, independent of time. Regardless of a system being observed at steady state, i.e., all state variables of a system are constant, there can always be flux of energy and entropy through the system (again at steady state), leading to a steady state entropy production, from the flux and its conjugate force (from the potential gradient) [2,8].

When the observational length-scales change from micrometer to that nanometer-scale, where rapid atomic vibrations are experienced, i.e., stochastic systems, the probabilities that various states will be repeated will remain constant at steady state. The energy and entropy of a stochastic system can be defined by a distribution. The oscillation frequency of the standing wave, times Planck’s constant, is the energy of the state, according to the Planck–Einstein relation. One of the characteristics of entropy is its extensive and non-conserved character. For a normal distribution, the entropy is maximized for a given variance of the distribution. A Gaussian (e.g., a random variable) distribution has the largest entropy amongst all random variables of equal variance, or, alternatively, the maximum entropy distribution under constraints of the mean and variance. Volume and microstates are related. From a microscopic standpoint, entropy can be linked to the probabilistic features of the accessible microstates of a system, i.e., to the peculiarities of the corresponding phase space. When the distribution is skewed (for example, because of a potential gradient), the entropy changes from that of the normal distribution over a volume of study. Each such distribution is associated with an entropy [9]. An entropy deficit, thus, can be associated when comparing a normal distribution to a skewed distribution and is called relative entropy or the Kullback–Leibler divergence [9].

At equilibrium, however, there is no entropy generation. Even when considering the stochastic scale for equilibrium conditions, there is no entropy production at equilibrium [1,8,27,28], although there are rapid vibrations or other forms of atomic scale movements. For systems at steady state, any gradient of potential causes entropy generation, e.g., a temperature gradient, a pressure gradient, a surface tension gradient, a chemical potential gradient, charge gradient, and so on [1,2,8]. There is a corresponding flux of energy associated with each gradient. This force-flux combination produces/generates entropy [1,8]. The generation of entropy has consequences for self-organization because a part of the entropy generation is related to the work’s potential loss, including the work that is required to maintain the self-organization features. This loss of work potential is often captured in shape formation (e.g., solidification microstructure patterns) [2]. The Gouy-Stodola theorem (named after two early proponents of thermodynamics) states that the exergy (work potential) destruction is proportional to the product of a reference temperature and d**S**gen/dt. Exergy is destroyed when entropy is produced, *but* that does not mean that there is no *effective* work done in the process. An amazing feature of entropy-producing, self-organization systems, particularly at steady state, is that a balance is struck between mechanisms which offer rapid entropy transport and entropy production.

## 3. Maximum Entropy Production Rate (MEPR) Principle

Based on the theoretical and experimental evidence, there appears to be an entropy generation principle that allows predictions for self-organization [2,3,10,11,12,13,16,17,18,19,20,21,75], i.e., for the selection of new patterns (morphologies), including the boundaries between the elements of the new shapes (patterns) that can evolve from a requirement of an optimal steady state to emerge. This principle is called the MEPR (maximum entropy production rate) principle.

The principle of MEPR states that, if there are sufficient degrees of freedom within a system, it will adopt a stable state, at which the entropy generation (production) rate is maximized within the control volume [1,2,3,4,5,6,7,8,9,10,11,12,13,14,15,16,17,18,19,20,21,22,23,24,25,26,27,28,29,30,31,32,33,34,35,36,37,38,39,40,41,42,43,44,45,46,47,48,49,50,51,52,53,54,55,56,57,58,59,60,61,62,63,64,65,66,67,68,69,70,71,72,73,75]. The pathway selections for mechanical, chemical, and morphological reactions are a consequence of this principle [2,3,9,10,11,12,13,14,15,16,17,18,19,20,21,22,32,72,75]. This principle is different from entropy maximization at equilibrium.

The application of this principle has been able to predict the planar-diffuse transition to cellular-like perturbations for solidification [13,75]. A critical test for the validity of this this MEPR principle, for a phase change system, is the successful prediction of diffusion constants during the transformation of liquid to a solid [13,75]. The principle is also predictive of faceted solidification to a planar diffuse state. Oscillatory conditions for patterns at an overall steady state in multi-pathway situations (e, g. several sequential sub-reactions for an overall chemical reaction) are also noted to occur often [1,12,31]. In such cases of self-organizing structures, it is possible to note pattern emergence and decay from decay-dissipative phenomena [30,31,32].

## 4. Patterns and Texture Examples from Directional Solidification, Wear, and Friction

In this article, two types of morphological rearrangement processes are considered for the study of self-organization with the MEPR principle. The first is directional solidification (discussed in this section) and the second is wear and friction (discussed in Appendix A). Directional solidification is a crystal growth method typically performed with the Bridgman or Choklarski techniques [23,24]. A liquid region is cooled and solidified (crystallized) by unidirectional heat removal. The crystallization occurs by an interface that moves in a direction opposite to the heat flow direction. The typical morphological features that are commonly noted are shown in Figure 1c.

**Figure 2:**
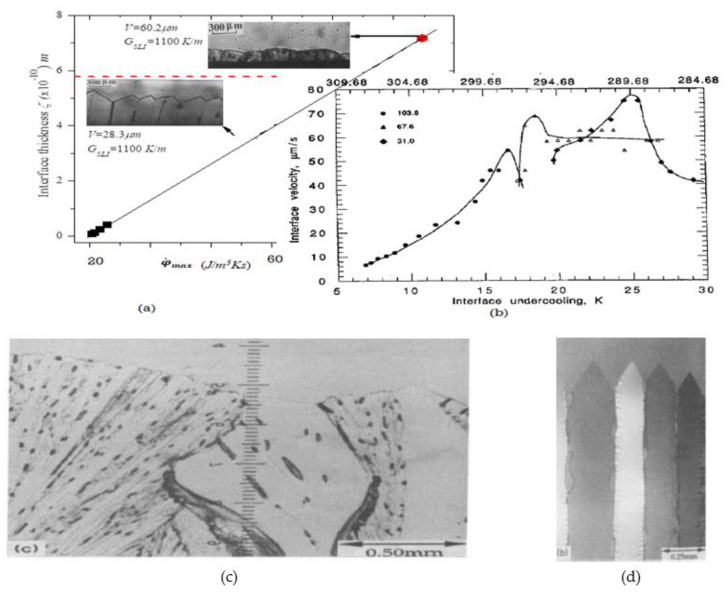
(**a**) A plot of the interface topography, as a function of the entropy generation rate for the solidification of salol (C_13_H_10_O_3_, produced by the interaction of salicylic acid and phenol), compared to predictions made by the MEPR calculation [13,75]. (**a**) The plot shows the transition from a cellular faceted morphology to non-facet morphology for salol, with increasing velocity (dotted black line). The horizontal dotted red line is the prediction [13,75] of the boundary between the facet morphology and non-facet morphology [41]. (**b**) Shows the facet morphological reorientation, with increasing driving force (velocity driven) [38,42]. This driving force, that establishes the entropy generation rate, scales with V/G or V.G [13,75]. Salol is an orthorhombic crystal structure, the (111) facet planes may take on 103.8, 67.6, or 31 degrees for the facet-tip angle. (**c**) Facet reorientation with increasing entropy generation. The higher solidification rates lead to the finer facet tips [42]. The faceted tip undercooling and the entropy generation rate (per unit volume) increase with the imposed velocity of solidification (growth), (**d**) from [42] the replacement of coarse tips by a finer tip, when required, with an increase in the velocity. As the solidification velocity is further increased, a side-branch formation feature is noted. This is a method of enhancing the entropy generation, as well as creating new defect-structures to enable entropy dissipation.

When an alloy melt is directionally solidified with an imposed positive temperature gradient at the transforming interface, a planar morphology is first noted at the solid-liquid interface for a low transition velocity (imposed independently in the experiment for a fixed temperature gradient and alloy composition). As the velocity is increased (e.g., by increasing the cooling rate), the planar interface becomes unstable to other shapes and transforms to a microscopically diffuse-interface, or to a macroscopically jagged/wavy cellular-shaped morphology, with several variations that are feasible in the topography [2,13,23,24,25,26,75]. The jagged variation is known as the faceted growth interface. All shape changes involve changes in the rate of entropy production per unit volume [2]. Figure 2 shows a schematic of an array of faceted columnar morphologies that can adjust their tip shapes to increase the entropy production rate as the velocity is increased. The figure shows the facet and secondary facet arm transitions for salol. The initial jagged facet feature of growth becomes unavailable for a higher imposed velocity. Figure 2 shows a plot of the interface topography, as a function of the entropy generation rate for the solidification of salol (aromatic powder, C_13_H_10_O_3_, produced by the interaction of salicylic acid and phenol) by the MEPR calculation [13,75]. The plot in Figure 2 shows the transition from cellular faceted morphology to non-facet morphology with increasing velocity, as shown by the dotted black diagonal line. The inset shows the various crystal orientations that the interface adopts to cope with the increasing entropy generation rate and entropy dissipation requirement. The interface velocity and interface undercooling (T_l_ − T_tip_) scale linearly, unless a new (111) configuration can replace the previous one [42]. Any increase in velocity results in the side-branch (SD) formation, which is a method of increasing the entropy generation [2]. This is a method of enhancing the entropy generation, as well as for creating new defects that can aid in entropy removal. Subsequently, non-faceted topographical forms, such as cells, can be noted [23,24,25,26]. Cellular and dendritic features may form with a further increase in the solidification velocity. These are the higher, entropy-producing variations [2].

**Figure 3:**
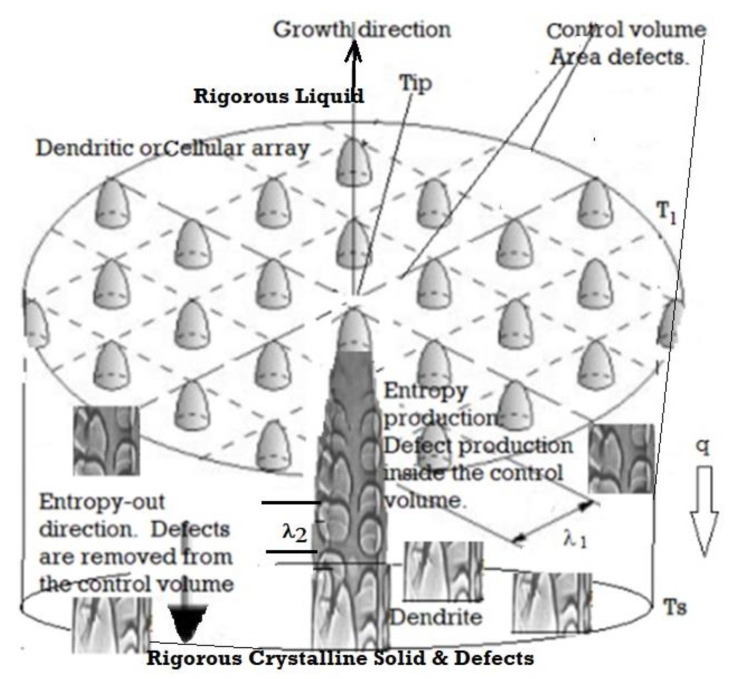
A schematic showing the directional solidification array features inside a control volume. The tip is at T_l_; the root is at T_s_. Only a few dendrites are shown in the mushy, solid liquid zone between T_l_ and T_s_. The control volume is defined as the region between the rigorous liquid and rigorous solid.

**Figure 4:**
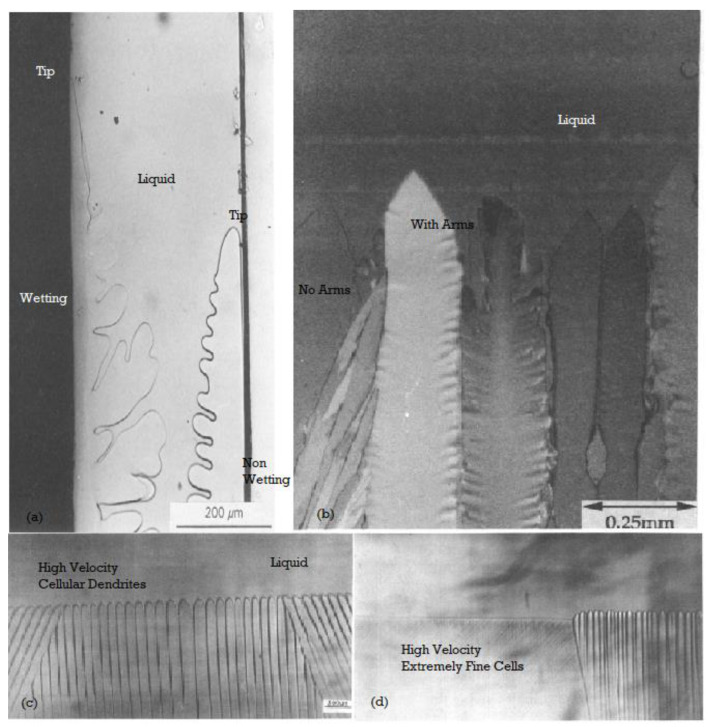
Transparent material solidification patterns from [23,24,35,36,37,38], pictured by moving the glass slide enclosure, containing the transparent material from a hot to the cold zone, thereby affecting crystallization. The solid-liquid zone (the control volume) is bound by the isotherms for T_s_ (Solidus or Eutectic temperature and T_t_ (tips temperature). The maximum work efficiency possible is (T_l_ − T_s_)/T_l_, i.e., when d**S**gen/dt = 0, alternately, the minimum work is when d**S**gen/dt is maximized. The minimum extracted work cannot be zero, because defects and curved interfaces form within the patterns. The materials studied are succinonitrile, SCN (C₂H₄(CN)₂ in (**a**), salol, (C_13_H_10_O_3_) in (**b**), and carbon tetrabromide, CBr4, in (**c**). In (**a**), the SCN is grown along wetting (low boundary entropy) and non-wetting interfaces (high boundary entropy) in the same experiment [37,38]. Note that the higher tip temperatures are associated with larger, confused structure and boundary regions of the secondary dendrites. In (**b**), from [42], the higher tip temperature is associated with sidearm-forming, faceted dendrites again; the higher, entropy-producing pattern require more defect area. As the demand on entropy production increases, because of a more severe imposed driving force (velocity of solidification), the solute partition function changes towards k_eff_~1. Consequently, very fine cells (i.e., with multiple boundaries), or a plane front (presumably with a large diffuse zone), re-emerge [36]. These are shown in (**c**,**d**), from [36]. Note in (**d**) that the transition from very fine cells to a more planar interface involves a drop in the interface temperature.

Figure 3 is a schematic of a columnar growth of dendrites. Figure 4 shows several unusual competing solidification features that can occur when the free solidification conditions are interrupted with physical surfaces or by a change to the driving forces [23,24,25,26,35,36,37,38,39,40,41,42,43,61]. The four variations and morphological transformations that are shown clearly indicate that a high velocity encourages higher entropy generation.

### 4.1. Low-Velocity Transitions, Facets to Smooth Curvature

It becomes very important to recognize diffuseness of the solid liquid interface, to understand the entropy production [2,13,75]. When diffuseness at the interface is encountered, the solidification is no longer a strict first order transformation. The diffuseness can comprise a considerable number of atomic layers, with a distribution of solid-like and liquid-like regions [13,75]. A solidification model, based on this MEPR principle, for interface instability, has accurately predicted the planar to facet (or planar (diffuse)) to a perturbed interface for several alloys, particularly when the solute partition coefficient is known [13,75]. The MEPR postulate can predict whether diffuseness or curved topographical features are most likely to form [2,3,13,75]. Note that minimum work cannot be zero because defects and curved interfaces form with the patterns, which implies that there is entropy production, based on the Gouy-Stodola theorem. This is a part of the work-potential loss (W_L_) during solidification.

The transition from an *atomistically*-smooth (faceted) to *atomistically*-rough interface (the onset of diffuseness) occurs when:

**V**/G_SLI_ = (√**N_c_**)·**d** (2)

**V** is the interface velocity, G_SLI_ is the temperature gradient in the diffuse region, and **d** is the atomic spacing normal to the growth interface. **N_c_** is the parameter that contains the partition coefficient and the liquidus slope, **c** indicates critical [13,75]. **N** (m^2^ K^−2^ s^−2^) is defined as $\left[\left(\frac{\;2\;\;\Delta h_{\mathrm{sl}}}{\;\Delta \rho_{k}{\;T}_{m}^{2}}\right)-\left(\frac{V\;\Delta T_{0}{\;R}_{g}\ln\left(\frac{1}{k_{eff}}\right)\;}{2{\;G}_{\mathrm{SLI}}{\;D}_{L}\;\Delta \rho_{k}{\;m}_{L}}\right)\right]$. Here, Δρ_k_ (kg·m^−3^), is the density change (shrinkage) between the liquid and solid.

Equation (3) can be developed and relates the maximum entropy production rate to the solidification variables by analyzing the driving force diffuseness [2,13,75]. Here, ${\dot{\varphi }}_{\max}$ (with a dot superscript) is the entropy generation rate per unit volume in the solid-liquid region (SLI) [13,75]. For a positive G_SLI,_ the entropy generation rate for low-concentration binary alloys can be expressed in the following manner [13,75]:

(3) $${\dot{\varphi }}_{\max}=\frac{V\;\Delta h_{\mathrm{sl}}{\;G}_{\mathrm{SLI}}\;}{T_{\mathrm{li}}{\;T}_{\mathrm{si}}}-\frac{\Delta T_{O}}{D_{L}}\frac{{\;V}^{2}R_{g}\ln\left(1/k\right)}{4{\;m}_{L}}$$

The subscript max., associated with the entropy generation rate, reflects the maximum that is feasible for that morphology [13,75]. We note that for a diffuse interface, the maximum entropy generation per unit volume can reduce significantly from its peak, when plotted as a function of the velocity [13,75]. Note that the first term in Equation (3) is ~$V\;\Delta h_{\mathrm{sl}}$/T_av_·ζ. As ζ is small (often lattice dimensions), the entropy generation rate per unit volume for a plane front interface is high. For alloys, T_i_ and T_s_ are the liquidus and solidus temperatures, ${\dot{\varphi }}_{\max}\;$decreases as T_li_ approaches T_l_. ${\dot{\varphi }}_{\max}\;\mathrm{is}\;$also further reduced by the second term in Equation (3). A peak $\mathrm{in}\;{\dot{\varphi }}_{\max}$ as a function of the imposed processing variables V or G is thus noted. For this condition, ${\left(\frac{\partial {\dot{\varphi }}_{\max}}{\partial V}\right)}_{\zeta ,{\;C}_{O}}=0$, which yields:

(4a) $$V/G_{\mathrm{SLI}}=(2/\Delta \rho_{k})({\dot{\varphi }}_{\max}/(Nc\cdot G_{\mathrm{SLI}}{}^{2\;}))$$

Or in terms of the imposed cooling rate, |V·G|,

(4b) $$V\cdot G_{\mathrm{SLI}}=(2/\Delta \rho_{k})({\dot{\varphi }}_{\max}/(Nc^{\;})$$

Equations (4a) and (4b) a predict the highest entropy generation conditions for stability. However, Equations (4a) and (4b) do not exactly pinpoint the transition to a different morphology. They indicate the peak value of ${\dot{\varphi }}_{\max}$. When comparing the plane front diffuse morphology with a wavy interface morphology (with diffuseness), the maximum entropy generation density rate criteria in the SLI for the onset of non-planar sinusoidal curved perturbations [13,75] is:

(5) $${\dot{\varphi }}_{\max}\cdot \;T_{\mathrm{li}}{\;T}_{\mathrm{si}}/(\;\Delta h_{\mathrm{sl}}{\;G}_{\mathrm{SLI}}{}^{2\;})\;\leq \;V/G{\mathrm{SLI}}_{\;}\leq \;2{\dot{\varphi }}_{\max}.\;T_{\mathrm{li}}{\;T}_{\mathrm{si}}/(\;\Delta h_{\mathrm{sl}}{\;G}_{\mathrm{SLI}}{}^{2\;})$$

A test for the model can be made by comparing the predicted solute diffusion coefficients at the critical conditions measured for interface breakdown [60]. The MEPR model has predicted reasonably accurate diffusion coefficients when comparing with independent diffusion coefficient measurements [13,75]. The MEPR model (Equations (4a) and (5)) is noted to have a better predictiveness, when compared to the MS model [44].

### 4.2. Medium Velocity Transitions: Cells, Cellular-Dendrites, and Dendrites

**Figure 5:**
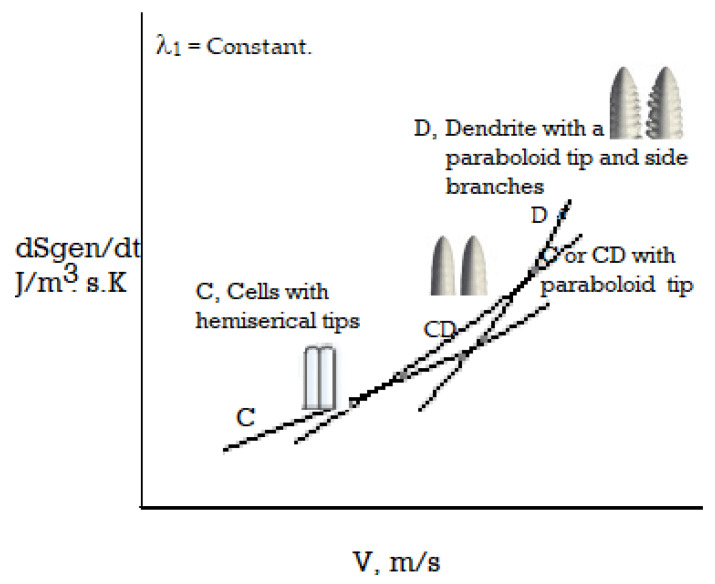
Entropy generation rate per unit volume for the three morphologies namely, Cell (C) (with hemispherical tip) to cellular-dendrite (CD) (no side branches but with a paraboloid tip) transition to dendrite (D) with side-branches and paraboloid tip. The tip temperature T_c__tip_ increases discontinuously for transition from a cell with hemispherical tip to cellular dendrite with the paraboloid tip. For the comparisons, λ_1_ is assumed to be constant.

Several morphological transitions that were not analyzed previously with MEPR, such as the cell to cellular dendrite to dendritic transitions, are discussed in this section. Arrays comprising of shapes such as cells, cellular-dendrites, dendrites, and others (shown in Figure 2, Figure 3, Figure 4 and Figure 5), can start producing entropy at a higher rate per unit volume compared to the diffuse plane front. Such morphologies and morphological change features change are discussed in detail below with Equations (6)–(12) to predict the morphology. The results are shown in Figure 5.

Dendritic, cellular, cellular-dendritic, and a few other odd-shapes (such as half cells, seaweed structures, and dendrites (shown in Figure 3 and Figure 4)) are also observed after solidification, depending on the processing conditions employed [23,24,25,26,42,43]. A dendritic array formed during directional solidification comprises of a regular array of paraboloid-tipped solid needles, with each dendrite displaying a somewhat periodic side-branch (SD) formation tendency. Cell-tips tend to be more spherical, compared to the more paraboloid dendrite tips. Cells do not have side-branches. Cellular-dendrites show dendrite-like tips but no side-branches. Cell-tip temperatures increase with velocity, whereas dendrite-tip temperatures decrease with velocity (for a fixed temperature gradient and alloy composition). The tip temperature (T_t_) of a dendrite, or the mean array tip temperature for a particular columnar morphology, lies between T_s_ and T_l_, where T_t_ depends on the imposed process conditions, namely the solidification velocity and temperature gradient (Figure 3 and Figure 4a). The spacings between individual dendrites, or the spacings of the secondary dendrite (SD) features, generally decreases with an increase in the solidification velocity. One of the important consequences of all such array structures is the occurrence of boundary defects between the cells and primary dendrites in the rigorous solid. These defects can range from low-angle to high-angle grain boundaries or even show new phase formation boundaries (because of microsegregation) between the dendrites [24,25]. The entropy production is also influenced by curvature and the enthalpy of transformation.

The amount of power employed for work (W) per unit time to create and maintain shapes and defects (which leave the control volume at T_s_) can be calculated from an energy balance, described by Equations (5) and (6):

(dW/dt) _max_ = −AV (∆h_sl_ − ∆h_m_) (6a)

where A is the area of the solidification interface normal to the DS growth direction. This work rate can also be, approximately, written as:

dW/dt = −A V [γ_gb_/λ_1_ + 6·γ_gb_·λ_2_/λ_1_^2^ + ω_D_] (6b)

The dendrite spacings λ_1_ and λ_2_ are the primary and secondary spacings, respectively. Here, ω_D_ is the defect energy associated with defect entropy ω_D_/Tav, not including boundary area defects but including energy/entropy terms associated with chemical species segregation, called microsgregation, and including the two-phase mixing of eutectics and solute gradients (for high alloy concentrations) that form between dendrites. W is the work done, in relation to W_L_, the loss in work potential. The sign in Equations (5) and (6) follow the standard thermodynamic conventions, where the work done on the system is positive. The symbol γ_gb_ is the energy of the solid-solid interface (grain-boundary) between the of cells or dendrites in the arrays. This energy can vary between ~1 mJ/m^2^ to 1000 mJ/m^2^ for metals, depending on the type of grain boundary or the microsegregated boundary [2,8,46]. A *steady state* [2] entropy balance gives:

d**S**gen/dt = −AV∆h_sl_(1 −T_l_/T_s_)/T_l_ + (dW/dt)/T_av_ (7)

d**S**gen/dt = AV∆h_sl_(1 − T_s_/T_l_)/T_s_ − AV [γ_gb_/λ_1_ + 6·γ_gb_·λ_2_/λ_1_^2^ + ω_D_]/T_av_ (8)

where T_av_ is a temperature between Ts and Tl. Here, Ti is the tip temperature for a generic morphology, whereas T_c__tip_ indicates cell tip temperature. Note that (1 − T_s_/T_l_) is the maximum work efficiency (Carnot efficiency) that is feasible for work between T_l_ (liquidus) and T_s_ (solidus). Figure 5 is a transition prediction, based on Equation (8), for cell to cellular dendrite and to a dendrite morphology, with an increase in the solidification velocity. Note that the second differential d^2^${\dot{\varphi }}_{\;}$/dV^2^ has a positive sign, when λ_1_ and λ_2_ are decreasing functions of velocity (a fact known known from experiments [23]), thus indicating that ${\dot{\varphi }}_{\;}$ is, indeed, the ${\dot{\;\varphi }}_{\max}$, when (d${\dot{\varphi }}_{\;}$/dV = 0).

If the entropy crossing the control volume boundary at T_s_ does not include curved interfaces, such as secondary dendrite envelopes, the entropy generation rates for cellular arrays (Equation (9)) and dendritic arrays (Equation (10)) may be approximated as:

d**S**gen/dt = AV∆h_sl_(1 − T_s_/T_c__tip_)/T_s_ − AV (γ_cell(__gb)_/λ + ω_D_)/T_av_ (9)

d**S**gen/dt = AV∆h_sl_(∆T_0_/T_l_)/T_s_ − A_SD_V (γ_dendrite(__gb)_/λ + 6·γ_gb_·λ_2_/λ_1_^2^ + ω_D_)/T_av_ (10)

The differentiation of Equations (9) and (10) with velocity, or the temperature gradient, indicates that λ_1_ will decrease with an increasing velocity and increasing temperature gradient, except under certain conditions of very high γ_gb_ or low T_av_. If the γ_gb_ changes abruptly with morphology, the spacing must also abruptly change between the scaling elements of a pattern that dissipates entropy. Assuming that the dendritic boundaries are of a much higher energy, this would imply that a cell(C)/cellular-dendrite (CD) to dendrite(D) transition is associated with an increased primary spacing at the transition, when plotted against velocity of solidification. This result agrees broadly with experiments [23]. When the secondary dendrites dominate the structure, a further approximation is possible:

d**S**gen/dt = AV∆h_sl_(∆T_0_/T_l_)/T_s_ − A_SD_V (γ_dendrite(__gb)_/λ + 6·γ_gb_·λ_2_/λ_1_^2^ + ωD)/T_av_ (11)

where A_SD_ is the surface area between the secondary dendrite features. Equating (9) and (10), and making the approximation that γ_gb_ is the same for both cells and dendrites, with the approximation that A_SD_~A, yields the elements of the connections (approximately) between the various self-organizing scales at the cell to dendrite transition-region and gives:

(λ_1_^2^/λ_2(C−D)_) = 6**Γ**·(T_s_/T_l_)/(∆T_0_ − ∆T_ctip(C−D)_) (12)

where ∆T_ctip_ is the cell tip temperature, minus T_s_. **Γ** is the boundary capillarity constant γ_gb_/∆s_sl_. Note that T_l_ (known from the phase diagram) becomes smaller with increasing alloying concentrations (when the solute partition ratio, k, is less than one). With increasing velocity (leading to an increased tip undercooling for dendrites), it is possible to calculate (λ_2(__C−D)_) or ∆T_ctip(C__−D)_) with Equation (12). The secondary arm spacing at the cell dendrite transition (λ_2(__C−D)_) is predicted to become smaller with increasing alloy concentration and with velocity, again in accordance with experimental observations [23,24,25]. As the T_ctip_ is experimentally found to be only slightly lowered with an increasing temperature gradient, the (λ_2(__C−D)_) is predicted to increases slightly with G_SLI_, depending on λ_2._ Note that the (λ_2(__C−D)_) is the secondary spacing, measured at T_s_ (and for conditions of the cell/cellular-dendrite to dendrite transition). Although numerous λ_2_ measurements are available for several alloys, there are very few experimental reports for (λ_2(__C−D)_). Regardless, some tests of the predictions can be made from published information.

**Figure 6:**
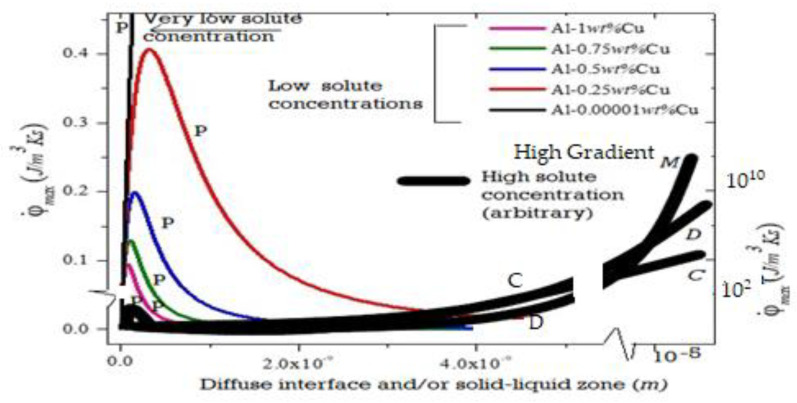
Maximum entropy generation rate per unit volume for planar diffuse interfaces (P) (thin colored lines), cells (C), and dendrites (D). M is a possible maximum entropy rate generation curve for a new featureless crystalline material or metallic glass. Note that the curves for C and D may show a peak, as per Equation (10), similar to the behavior of the plane front with diffuse interface (P). The P (Plane Front) plots are from reference [13,75]. The M indicates metallic glass formation made by splat or ribbon cooling methods.

For a nickel-based aircraft alloy-IN-718 [26] and alloy Rene-108 [61], the (λ_2(C−D)_) is noted to be about ~100 micrometers. λ_1_ at the C-D transition condition is reported to be ~300 micrometers for both alloys. The ∆T_0_ is 369 K for the Rene-108 multi-component alloy but only about ~30 K for IN-718 multi-component alloy [61]. Therefore, the tip temperature differences of the cell/cellular-dendrite and the dendrite at the C-D transition should be very small (as noted) for a meaningful prediction by Equation (12). This also appears to be the general experimental finding with all metallic alloys [24,25,43], thus, further indicating that the entropy maximization principle may be employed as a key selection criterion [2,3,13,75] for the prediction of specific patterns during solidification. Figure 5 and Figure 6 are plots of the entropy generation rate per unit volume for several commonly identifiable solidification morphologies. 

### 4.3. High-Velocity Regimes Including Featureless Solids and Metallic Glass

In the very high solidification velocity regimes, morphological features like very fine cells, featureless crystalline plane fronts and even frozen liquid (such as metallic glass) are noted [24,25,40,44]. There is one prediction made by the MS model [44] which is not completely borne out by the MEPR formulation, namely, the emergence of a high-velocity plane front (called the absolute stability condition in the MS model) *without* altering the partition coefficient. In the MEPR model, the solute partition coefficient, k, must increase for an alloy that shows a negative *slope* of the liquidus (m_l_) and solidus (m_s_), to establish a plane front at very high interface velocities. This is not necessarily so for the MS model. The experimental results by Trivedi et.al [36] and Sekhar [45] do show extremely fine cells, and even a planar interface at very high growth rates for low solute concentration alloys; however, the partition coefficient cannot be ascertained from the two reports, thus making it difficult to compare the MS and MEPR models in this high-velocity growth regime.

When the d**Se**/dt is large (for example with rapid heat removal at the CV boundary) but d**S**gen/dt from a particular morphology is inadequate, then the phase itself could alter to reestablish a steady state (e.g., metallic glass can result, instead of a crystalline solid). For freezing into a crystalline form, the typical entropy generation rate mechanisms are captured by crystal structure and shapes with defects, i.e., by crystal structure, reorientation and expansion, interface diffuseness, cell/dendrite patterns, or by creating a new phase (crystalline or glassy) [2]. For the freezing of a liquid into a glass, there is no need to invoke segregated area defects in the solid [36,37]. Note, in Figure 4d, that the transition from very fine cells to a more planar interface involves a drop in the interface temperature. This is required in MEPR but not required in the MS model.

For a featureless phase to form, in lieu of a crystalline phase with boundary-defects, a very large amount is work could be required, i.e., a highly diffuse mixed interface [2] or a glassy phase, where there is no latent heat release (in a sense resembling a second order type of reaction transformation). Equations (1) and (3), are easily satisfied at steady state for both a wide diffuse interface with the partition coefficient k tending to 1 or ∆Co = (Cl* − Cs*) = 0 [2,44] or for glass formation. For a pure metal, or when k tends to 1, the highly diffused interface could produce significant amounts of entropy as the diffuse region is extended, but for an alloy, the entropy generation per unit volume peaks with the extended, large, diffused interface [13,75].

For metallic glass formation, ∆h_sl_~0, the entropy generation is from the steep and non-linear temperature gradient over a very small thickness. For the control volume, the boundaries are at the T_l_ and T_g_ (glass transition temperature). Below T_g_, the rate of volume change for glass is very low with further cooling. The supercooled liquid (not yet a glass) generally has the coefficient of thermal expansion of a liquid. The faster the cooling rate, the higher is the molar volume and the molar enthalpy of the glass (solid) that forms. The rates of entropy generation per unit volume for a featureless crystalline feature phase (Equation (13)) or for a glassy phase (Equation (14)) are respectively:

(13) $${\dot{\varphi }}_{\;}=K_{\mathrm{av}}{G_{\mathrm{SLI}}}^{2}/(T_{\mathrm{av}}{)}^{2}-∆\mu_{\mathrm{sl}}\cdot V/(\zeta \cdot T_{\mathrm{av}}\cdot \nu_{m})$$

(14) $${\dot{\varphi }}_{\;}=K_{\mathrm{av}}\;(Tl_{\;}-T_{g}{)}^{2}/(\zeta_{g}\cdot T_{\mathrm{av}}{)}^{2}=K_{\mathrm{av}}{G_{l}}^{2}/(T_{\mathrm{av}}{)}^{2}$$

where K_av_ is the thermal conductivity (of the diffuse interface in Equation (13) and of the supercooled liquid becoming solid- glass in Equation (14)). T_g_ is the glass transition temperature and ζ_g_ is the zone thickness between T_l_ and T_g_. The very first metallic glass was made with a imposed cooling rate of ~10^6^ K/s [40] giving ${\dot{\varphi \;}}_{\;}$~4 × 10^10^ (J/m^3^·K·s), based on a gradient of 10^6^ K/m. Figure 6, shows an entropy generation rate per unit volume plot that includes metallic glass formation. Note that, in such severe conditions, extremely small thicknesses could experience a high temperature gradient because of a high Biot number with a high heat transfer coefficient. (The Biot number is the ratio of the thermal resistances *inside of* a body and *at the surface* of a body). Biot numbers much larger than 1 indicate a temperature gradient in the splat cooled material.

Equation (14) could indicate that a metallic glass would be a common occurrence for small thicknesses that is cooled at a very rapid rate; however, a comparison with Equation (3) shows this may not be the case where a highly diffuse interface is possible, which, because of the high Δhsl, heat of fusion (Jm^−3^) would be able to generate a much higher entropy (during cooling) for the same thin dimensions. In contrast, materials with a low heat of fusion per unit volume, such as glass-forming silicate ceramics, the glass formation is the preferred pattern morphology (atomic configuration) during a cooling process. 

### 4.4. Range of Solidification Morphological Transitions

The maximum work efficiency possible is (T_l_ − T_s_)/T_l_, i.e., when d**S**gen/dt = 0. Minimum work is when d**S**gen/dt is maximized [2]. The minimum extracted work cannot be zero, because defects and curved interfaces can form within the patterns. 

**Figure 7:**
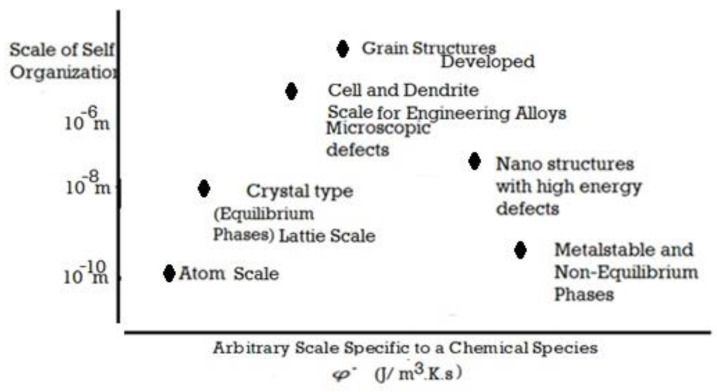
The scale of the typical features and patterns for self-organization in a solidified material, as a function of the rate of entropy generation per unit volume during transformation from liquid to a solid.

Crystalline patterns or a plane front instead of glass (or vice versa) are, thus, simply a matter of allowing entropy production rate per unit volume to be maximized in the control volume of the transformation. This is shown in Figure 6. The largest scale for the self-organized patterns appears to be influenced by the entropy that must be produced at a rate that can keep pace with the dissipation demand. Figure 7 is a schematic of the rate of entropy generation that affects the scale of new pattern formation. It appears that this is also the scale of defect distribution relevant to yield optimal utility to the scale of the patter or array (see Figure 1b).

## 5. Discussions: The Utility of Self-Organization

A general characteristic of self-organizing systems is that they are robust or resilient (i.e., stable) [1,2,3,4,5,6]. This means that they are relatively insensitive to other self-organizing perturbations and have a strong capacity to restore themselves.

**Figure A1:**
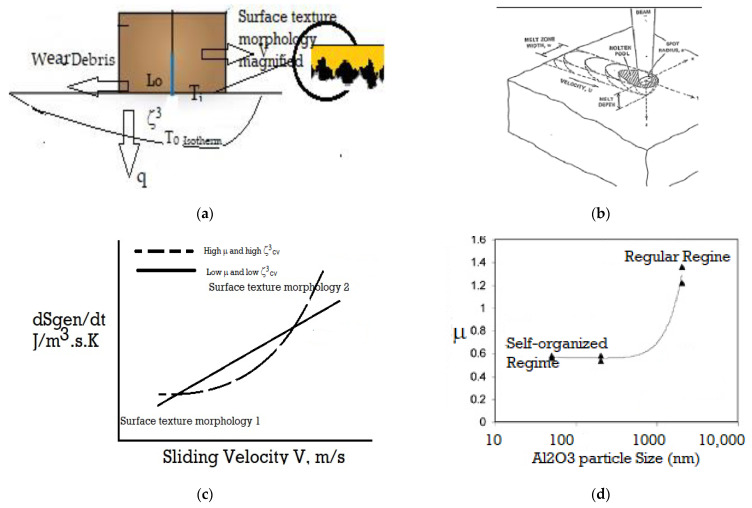
(**a**) Schematic of a body moving with a velocity V, over a surface (friction pair). Entropy is generated in the control volume ζ^3^ (m^3^) at the friction interface, which includes the Hertzian zone, i.e., the zone of contact of the two surfaces (the roughness in this zone is shown in the magnified inset). The control volume is bound by T_i_ and T_o_. The entropy leaves the control volume with the heat q at T_o_ and with the wear debris T_i_, respectively. The approximate entropy generation rate, as a function of the velocity (of sliding) for two different morphologies at the interface, is characterized by the asperity roughness height and the autocorrelation length; (**b**) the thermal problem is similar to an area source moving on a semi-infinite substrate [56,65,66], for which a schematic is shown, T_0_ is the ambient temperature; (**c**) a plot of Equation (A6) for d**S**gen/dt, as a function of the sliding velocity for two different surface morphologies, 1 and 2, corresponding to two different coefficient of friction values (μ) for the solid-solid pair; (**d**) a plot showing extremely low friction for a self-organized surface asperity cluster, compared to other forms of surface features, modified from the data reported in Reference [54].

There is utility to self-organization in chemical and metallurgical systems. Appendix A discusses entropy generation and the coefficient of friction within the framework of MEPR and surface texture. Self-organized patterns of surfaces have been shown to display the lowest friction and wear [54,71] (see Figure A1d in the Appendix A), the lowest friction coefficients [56], and improved toughness [57].

For stable self-organized configurations, Equation (1) (with d**S**cv/dt = 0) is satisfied, i.e., a steady state is reached. With new patterns, a changed manner of energy and entropy fluxes become operative, as noted in the previous section and Appendix A. Work can also manifest during the energy exchange, which leads to ordering. This work, in turn, can be used to build better and more efficient features for the production and transport of entropy. The coordination in self-organized systems, seemingly arises out of the local interactions between smaller-sized, component–parts of a system, which can quickly, otherwise, disorganize if not ordered into a new pattern. The process and the rate of self-organization can be “spontaneous” [14], i.e., it is not necessarily controlled by any auxiliary agent outside of the system. It is often triggered by random fluctuations that are amplified by positive feedback, which allow maximum entropy production and provide a method for entropy storage and transport. This becomes the basis for controlled defect formation events. The resulting organization is, thus, in a sense, wholly decentralized or distributed, yet entangled over all the components of the system.

**Figure 8:**
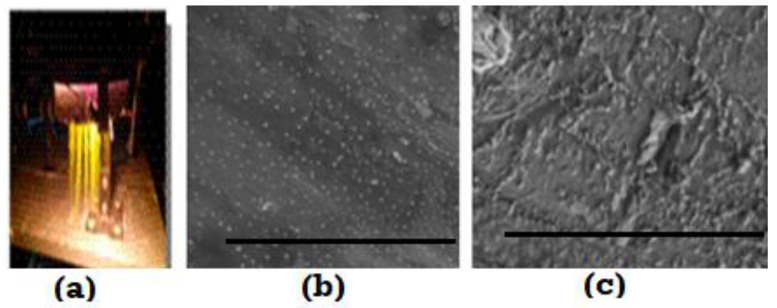
(**a**) A self-organized efficient, one atmosphere plasma (from www.mhi-inc.com accessed on 26 July 2021). The plume length is about 800 mm. When this plasma interacts with a tool-bit surface, self-organized asperities form as a result of surface chemical reactions, as shown in (**b**,**c**). (**b**) Gaussian-like (affine) distributed random asperities on M35 tool steel surface are shown (scale bar is 3 microns). In (**c**) Quasi R (Quasi R is a trademark of MHI Inc., Cincinnati, OH, USA) asperities, which deviate from Gaussian distribution of RMS heights are shown (scale bar is 3 microns). Limited measurements for the coefficient of friction indicate that the texture in (**c**) has a very low coefficient of friction [56]. The asperities in (**b**,**c**) are comprised of a nanoscale phase distribution of extremely fine nanoscale iron oxides and iron nitrides that display high elastic modulus along with a high hardness. The Vickers of the Hertzian zone is Hv~1300.

There are some reports that suggest that self-organization is a process enabler for various optimizations. An example is a laser or plasma beam interaction with a surface that leads to self-organization in surface features [54,57,71,72]. Self-organized structures noted in some plasmas (Figure 8) show non-extensive and non-Gaussian character. One example, is an extremely efficient plasma, called the E-Ion plasma (The E-Ion plasma is a product of MHI Inc. Cincinnati, OH, USA. www.mhi-inc.com, accessed on 26 July 2021). (Picture used with permission.) [56]. The formation of such a plasma is thought to involve multi-scale, strong interactions across the scale of fermions and bosons, to the macroscopic level of plasma-streams, which lead to organized patterns of distributions of the activated species that further enable organized asperities on the surface of a metal from the plasma/metal interactions (Figure 8). Such surface textures can give rise to extremely low friction coefficient displaying surfaces (see Appendix A). Inside the asperities, extremely fine, nano-scale chemical mixtures of iron oxides and iron nitrides influence patterns at a larger size/scale of the surface asperities (by mixing at a nanoscale with recognizable patterns that are larger, by about one magnitude, than atomic clusters).

There are other examples that highlight the utility of self-organized patterns. In the information–communication literature, where conventional entropy is replaced with the Shannon entropy concept, a self-organization is a negentropy feature that allows for ordering and, thus, utility. The ordering that is associated with pleasant sound patterns (pressure wave and shock wave patterns) and their related boundaries leads to a communicative language. In nonlinear dynamical systems, the evolution of entropy is a linear function of time or equivalently the entropy production rate is constant (a feature of steady state), known as Kolmogorov-Sinai entropy [48]. It is also known that variations in the rates of entropy generation with time are possible when the geometry of the phase space is fractal [49,52,72,73]. 

Self-organized structures, whether crystal structures, flower patterns, or fungi (including mushrooms), that bear a relationship to the Fibonacci series [57] are evolved from another pattern or an older state. during a dynamic process, *albeit* sometimes very slowly. In both living and inanimate systems, self-organization leads to significant optimization and often to a lowering of the resources or energy required to carry out a process.

The formation of self-organized structures, particularly in live biological structures, appears to demand minimal power. This is probably what allows several neurons to form in a human brain [12,53,58,59], without a need for significant energy demand. Network organizations, during certain evolutionary periods, is possibly a result of self-organization events. Note that the entropy generation rate per unit volume of cellular structures for human and chemical solidification cell-evolution (discussed in Part 4) are of the order of ~1 (J/m^3^·K·s), assuming ~O(10^3^) J/K of entropy is dissipated by the human brain (volume ~10^−4^ m^3^) over a year, during its growth/development stages [13,58,59,75]. The structure of the cells in the human brain [53,58,59] do appear to have resemblances to the defect-enveloped, entropic pathways envelopes seen in microsegregated solidified grains, cells, or dendrites [8,23,24,25,26,61]. Regardless, more studies that are warranted to establish the similarities of shape evolution between inanimate solidification studies and human cell development particularly because biological cell-multiplication and grain-nucleation happen by vastly differing mechanisms for chemical transport.

Human metabolic rate and shape formation, at several length scales of interest, are seemingly correlated. An increased metabolic rate, along with changes in energy allocation, is seen in the evolution of human brain size and evolutionary history, [76] which is a possible reason for self-organization during evolution. Self-organization in biological systems [12,56,69] is perhaps an answer to an environmental change, for which the existing system cannot cope. Thermodynamic efficiencies in self-organizing systems are related to the work extracted because of a change in the control parameters. They peak at a critical point for a particular shape or discontinuous shape transition [13,62,72,75]. For living and dreaming brain-systems, one result of a self-organizing process is that a new ‘self’ is possibly created [51,53]. The brain activity is thought to self-organize into patterns that can lead to different consciousness from thought processes, as well as a different arrangement of images that lead to innovative thought process [51,53,58,59].

There appears to be a specific, self-organized structure that is ‘natural’ for optimizing critical properties at various length scales, e.g., ordered crystalline structures are routinely used as high-performance engineering alloys when strength, ductility, and fracture toughness are of critical importance (or flower petal-patterns that optimize sunlight or the volume available for growth). This stability and maintenance of a morphology involves continuous work.

An orderly pattern is formed because it is the most entropy-producing structure per unit volume. In the two examples discussed in this article for solidification and wear, we have noted that self-organization is associated with a control volume. There is a specific volume of interest that is associated with a repeating pattern-element and the associated boundary defects that change abruptly during a self-organization type of morphological transitions. There is, thus, possibly a morphological “equilibrium”, which provides optimal stability for a self-organized structure over other competing shapes *in each environment* [2,74]. It is likely that only after a process decay from steady state conditions, does the free energy equilibration become the dominant principle for selection. In this article, we have also considered boundaries that envelope shapes that are important for entropy generation, entropy dissipation, and self-organization. Such boundaries (the defect-regions) are possibly extremely important to establish patterns and influence the export of entropy.

## Data Availability

Not applicable.

## Nomenclature and Abbreviations

Letter Symbols	
A	area of an interface in a solid-liquid region (m^2^)
A_SD_	surface area of the secondary dendrite features
d	interplanar lattice spacing (m)
∆C0	change in concentration for the liquid from the position of rigorous liquid to the bulk. ∆Co = (Cl* − Cs*) where Cl* and Cs* are the composition of the local rigorous-liquid and solid, respectively
D	diffusion coefficient (m^2^·s^−1^)
f	facet
F	is the friction force (kg·ms^−2^)
G_SLI_	gradient across a diffuse interface (K·m^−1^)
G_l_	temperature gradient in the liquid or supercooling liquid (K·m^−1^)
Δhm	heat of fusion of a solid with defects (J·m^−3^)
Δhsl	heat of fusion (J·m^−3^)
H	hardness (J·m^−3^ or MPa)
Js	solute flux in a liquid entering a solid-liquid interface (mole·s^−1^)
k	equilibrium partition coefficient obtained from the phase diagram (dimensionless)
keff	effective partition coefficient at a solid-liquid interface (dimensionless)—a function of the velocity and diffuse interface structure
ΔKE	gain or loss in kinetic energy (J)
K_L_	thermal conductivity for a rigorous liquid (J·m^−1^·K^−1^·s^−1^)
K_S_	thermal conductivity for a rigorous solid (J·m^−1^·K^−1^·s^−1^)
K_av_	average thermal conductivity in the control volume (J·m^−1^·K^−1^·s^−1^)
K_wear_	wear constant, dimensionless ~10^−4^ for metal pairs.
Lo	sliding distance (length of object in Appendix A, in the direction of travel)
m_L_	slope of the equilibrium liquidus at the SLI for a binary material (K·m^3^·mole^−1^)
m_s_	slope of the equilibrium solidus line at the SLI for a binary material (K·m^3^·mole^−1^)
MEPR	maximum entropy production rate per unit volume (J·m^−3^·K^−1^·s^−1^)
MS	Mullins and Sekerka criterion [44]
N_p_	normal force (kg·m·s^−2^)
nf	non-facet
Rg	molar gas constant (J·mol^−1^·K^−1^)
R_RMS_	RMS height (m)
**S**f	Entropy flux rate (J·K^−1^·s^−1^) to and from a solid-liquid interface with its surrounding
d**S**gen/dt	entropy generation rate in a diffuse region (J·K^−1^·s^−1^)
d**S**in/dt	rate of entropy entering a control volume (J·K^−1^·s^−1^)
d**S**out/dt	rate of entropy leaving a control volume (J·K^−1^·s^−1^)
d**s**gen/dt	entropy produced/generated rate density (J·m^−3^·K^−1^·s^−1^)
S_LG_	entropy generation rate density by the solute gradient in a liquid (J·m^−3^·Ks^−1^)
(**S**gen)*max*	maximum entropy generation (J·K^−1^)
d**S**cv/dt	total steady state entropy rate in a control volume (J·K^−1^·s^−1^)
dscv/dt	total steady state entropy rate density in a control volume ((J·K^−1^·s^−1^·m^−3^)
t	time (s)
SLI	solid-liquid interface
SD	side-branch secondary structures, such as secondary dendrites.
q_1_/τ	the heat transfer rate to the main body in a friction pair.
Tli	liquidus interface temperature at a rigorous liquid interface (K); generally assumed to be T_l_ (the equilibrium liquidus temperature)
Tsi	solidus interface temperature at a rigorous solid interface (K); generally assumed to be T_s_ (the equilibrium solidus or eutectic temperature)
T_ctip_	tip temperature of a cell
T_tip_	tip temperature of cells, dendrites, or facets in an array
T_i_	friction interface temperature of a friction pair
T_0_	room temperature for the friction problem
T_i_	friction interface temperature of a friction pair
Tm	melting temperature (K) of the pure metal or species
Tav	average temperature between Tli and Tsi in diffuse interface or solid-liquid region (K), when G is negative or close to zero
T_0_	room temperature (ambient)
ΔTSLI	temperature difference across a solid-liquid interface (K)
ΔT_ctip(C−D)_) = T_ctip_ − Ts) (K)	at the cell or cellular dendrite transition to a dendrite
(dclG/dz) or (ΔCO/δc)	change in solute gradient in a liquid (mole·m^−4^)
ΔTO	solidification temperature range (K) = −mL·ΔCo(1/k − 1)
ΔTi	temperature difference between rigorous liquid and rigorous solid (K) = T_l_i − T_si_
ΔTctip	(T_ctip_ − Ts) (K)
V	solidification interface velocity (ms^−1^)
V	sliding pair velocity (ms^−1^) (see Appendix A)
WL	lost work (J)
W	work (J)
Greek symbols	
ΔΩS	volume shrinkage (m^3^)
β	the autocorrelation length (m) of the surface
|Δρk|	density shrinkage (kg·m^−3^)
ρl	density of rigorous liquid (kg·m^−3^)
ρs	density of rigorous solid (kg·m^−3^)
γ_gb_	solid-solid boundary energy (e.g., between primary cells or primary dendrites)
λ_1_	primary spacing at the Ts isotherm
λ_2_	secondary spacing at the Ts isotherm
λ_2(C−D)_	secondary spacing at the Ts isotherm for the conditions of cell, to dendrite transition
Δμc	chemical potential difference (J·mole^−1^)
**Γ**	boundary capillarity constant γ_gb_/Δs_sl_.
μ	coefficient of friction
θ	fraction of energy transferred to heat.
ζ	solid-liquid interface thickness (m), i.e., the diffuse interface thickness
ζ_g_	is the zone thickness between T_l_ and T_g_
ξ	wear volume
τ	(length of travel, Lo)/V (s)
ζ^3^_cv_	thermal control volume for steady state friction (Appendix A)
ω_D_	energy of defects (J·m^−3^) other than area defects
${\dot{\varphi }}_{\;}$	maximum entropy generation rate density for a moving interface (control volume) (J·m^−3^·K^−1^·s^−1^)

## Appendix A. Self-Organization of Surface Texture during Friction and Wear

We examine the MEPR principle, for studying the self-organization behavior of a pair of contacting surfaces subjected to solid-solid friction and wear. A particular morphology of asperities or surface texture will determine the coefficient of friction [54,55,56]. The texture morphology is defined by the surface-characteristics, such as the RMS height (R_RMS_) and the autocorrelation lenght (β), of a rough surface [56]. When there is stability of the coefficient of friction, all the defining features of the asperities, such as the RMS height (R_RMS_) and the autocorrelation length (β), decay exponentially at the same rate [56], thus preserving the steady state, regardless of the size of the asperities, i.e., wear does not necessarily alter the coefficient of friction (at least in certain regimes of wear).

Consider the dry friction interaction of an object moving on a surface (Figure A1a). Amonton’s friction law relates the normal load N_p_ to the reactionary friction force F that must be overcome for pushing the object with a certain velocity; the law is written as F = μ N_p_. Here, μ is the coefficient of friction [56]. The maximum heat generation rate, q/τ, is given by [63]:

q/τ = μ·V·N_p_ (A1)

Here, V is the steady state sliding velocity. Archard’s equation [63,64] for wear relates the wear volume ξ, to the normal load N_p_, the sliding distance S, and the hardness H, through a proportionality constant K_wear_, i.e., the wear coefficient [63,64,65]:

ξ = K_wear_·SN_p_/H (A2)

During sliding, only a portion of friction energy is however converted to heat and the remainder results in plastic deformation, micro-cracks, and a change in surface roughness. The heat energy is also linearly correlated with the wear volume during sliding wear [63]. The wear volume rate per unit area (in the Hertzian zone) dξ/dt is thus:

dξ/dt = K_wear_ (q_1_/τ)/ημH (A3)

H is the Hertzian hardness [56,63,64,65]. τ is the time equal to Lo/V, where Lo is the length of the sliding object. The value of the wear coefficient is K_wear_~10^−3^ to 10^−4^ (dimensionless) for many metal pairs [63] (note that this term incorporates the Elastic Modulus/Hardness ratio to calculate the plasticity coefficient, plastic deformation, and heat release). The wear volume ξ is not the control volume for the entropy balance problem. The control voulme (ζ^3^_cv_) is the volume which is bound by the isotherms T_0_ (room temperature) and the T_i_ (the contacting interface temperature). *Note that the entire Hertzian zone should be considered inside the control volume to apply MEPR.*

The heat partitioning factor can be used to assess the heat distribution [63]. The heat transfer rate to the main body is q_1_/τ:

q_1_/τ = ημVN_p_θ (A4)

where θ is the fraction of energy that is transferred to heat (About 85% in most machining operations.). The heat generation establishes T_i,_ the interface temperature. η is the heat partitioning constant between the solid pairs. If the sliding object is small, the object temperature is also T_i_, with η~1.

The thermal conditions for the friction problem are analogous to the heat flow problem of an area heat source moving on a surface (e.g., a moving electron or laser beam for surface modification). At steady state, for an area heat source (with a constant heat flux on a surface and travelling with a velocity V on the surface) a steady state stationary volume (ζ^3^_cv_) is established, bound by the T_0_ (room temperature isotherm). From references [66,67], we note that this steady state volume (ζ^3^_cv_) decreases with increasing velocity for a fixed-power heat source. Applying the steady state entropy equation [1] the entropy generation rate per unit volume inside a control volume can be calculated. If the entropy exchange by heat transfer leaves the body at the isotherm T_0_ (the ambiant temperature), and that the entropy associated with the wear debris leaves at T_i_, the entropy balance may be written as:

d**S**gen/dt = (q_1_/τ)/T_0_ + ω/Ti = ηθ μV N_p_/T_0_ + ω/Ti = ∫_cv_ K·(ΔT/T)^2^ dζ^3^ + ω/Ti (A5)

Where the term ω contains the energy of the deformed and loose debris. The term ω/T,_i_ is thus the entropy that exits the control volume with defects/wear debris, i.e., the part that does not leave with the heat to the semi-infinite substrate. The MEPR principle uses the unit volume approach for the comparison between competing morphologies. The entropy generation rate per unit volume for a particular surface morphology at the region of contact (see Figure 1c and Figure A1a) at steady state is thus:

d**S**gen/dt = (q_1_/τ)/T_0_/ζ^3^_cv_ + ω/Ti/ζ^3^ = ηθμV N_p_/T_0_/(ζ^3^_cv_) + ω/Ti/(ζ^3^_cv_)
 = (∫_cv_ K·(ΔT/T)^2^) dζ^3^)/(ζ^3^_cv_) + ω/Ti/(ζ^3^_cv_) (A6)

Note that T_i_, the contact temperature (interface temperature), is set by the coefficient of friction and material properties that control deformation of the surface features during the sliding process [56,69,70]; ζ^3^_cv_ is the control volume. Therefore, for a fixed surface-asperity configuration (i.e., morphology) at steady state, there is a fixed entropy generation rate, which increases with velocity, for a fixed dimension of the control volume. (Equation (A6)). When the control volme dimensions change [66,67] because of the severity of the heat produced, then correspondingly, the entropy generation rate per unit volume also changes, which could lead to inversions, shown in Figure A1c. Consequently, the morphology is altered by wear processes to one which displays a lower coefficient of frction. Equation A6 is simple but powerful application of MEPR to wear surface texture.

Figure A1a is a schematic of a body moving with a velocity V, over a surface (the body and substrate form a friction pair). Entropy is generated in the control volume (ζ^3^ m^3^) (i) at the friction interface, which includes the Hertzian zone, i.e., the zone of contact of the two surfaces (ii) by the thermal gradients. The control volume is bound by T_i_ and T_o_. Figure A1b shows the similarity of the heat transfer problem to one where an area source moves over a semi-infinite substrate. Figure A1c is a plot of the entropy generated per unit volume, for the comparison of two different friction coefficient of pairing materials (following Equation (A6)). Note that two lines are shown, representing two different scenarios of surface texture, i.e., for the two different coefficients of friction. The different surface morphologies are defined by the RMS height (R_RMS_) and the autocorrelation length (β) [56] of the surface texture (asperities). Note, again, that the surface texture features of interest (Figure A1a) are located inside the control volume, as only then is the MEPR applicable [73].

Assume that the entropy generation rate is at its maximum in the control volume for steady state conditions. The MEPR principle indicates that the lower coefficient of friction displaying morphology can produce/generate more entropy (per unit volume and time), depending on the sliding velocity [56] and other material parameters. This is shown in Figure A1c. Self-organization has been noted to occur and modify surface texture as the velocity is increased for an asperity morphology that can yield a lower coefficient of friction with increased velocity, while transitioning to a lower coefficient of friction [54,56,68,70]. Figure A1d is plot showing measured low friction coefficient for a self-organized surface asperity cluster, compared to other forms of surface features [54]. It should be noted that the precise relationship between the surface parameters and the coefficient of friction is not yet fully established [56,69,70]; however, Equation (A6) provides a framework to discuss the various perplexing texture morphology transitions that are observed during frictional wear. Figure A1c, could for example explain why polishing of a surface may sometimes lead to higher, or sometimes to a lower, roughness, depending on the velocity of the polish, an observation noted in reference [56]. Equations (A6) and Figure A1c indicate that a change in velocity can lead to an increase or decrease in the coefficient of friction [56].

It should be noted that, unlike the case of solidification discussed in the main body of this article, the transition during wear to a different steady state surface texture morphology by self-organization could be more complicated because of the numerous surface texture possibilities. The required new entropy generation pathway may not be available unless the wear(ing) conditions are able to transition through several steady state regimes for creating a new surface texture pattern (morphology). In fact, prior to the establishment of a steady state, there may be forces that cause wear to occur in a manner that causes “settling-in” before an effective steady state is achieved, with a particular texture. This settling-in is common to many friction pairs, in both inanimate and biological traction pairs [54,56,70,71]. Settling-in always appears to yield a lower kinetic coefficient of friction. Similarly, the type of wear at the contact interface could be influenced when transitioning from one surface-morphology to another, to increase the entropy generation rate per unit volume inside the control volume (Figure A1c).
